# Implementing Medical Technological Equipment in the OR: Factors for Successful Implementations

**DOI:** 10.1155/2018/8502187

**Published:** 2018-08-29

**Authors:** Navin Sewberath Misser, Bas van Zaane, Joris E. N. Jaspers, Hein Gooszen, Johan Versendaal

**Affiliations:** ^1^HU University of Applied Sciences, Utrecht, Netherlands; ^2^University Medical Center Utrecht, Netherlands; ^3^Open University, Heerlen, Netherlands; ^4^Radboud University Medical Center, Nijmegen, Netherlands

## Abstract

Operating rooms (ORs) more and more evolve into high-tech environments with increasing pressure on finances, logistics, and a not be neglected impact on patient safety. Safe and cost-effective implementation of technological equipment in ORs is notoriously difficult to manage, specifically as generic implementation activities omit as hospitals have implemented local policies for implementations of technological equipment. The purpose of this study is to identify success factors for effective implementations of new technologies and technological equipment in ORs, based on a systematic literature review. We accessed ten databases and reviewed included articles. The search resulted in 1592 titles for review, and finally 37 articles were included in this review. We distinguish influencing factors and resulting factors based on the outcomes of this research. Six main categories of influencing factors on successful implementations of medical equipment in ORs were identified: “processes and activities,” “staff,” “communication,” “project management,” “technology,” and “training.” We identified a seventh category “performance” referring to resulting factors during implementations. We argue that aligning the identified influencing factors during implementation impacts the success, adaptation, and safe use of new technological equipment in the OR and thus the outcome of an implementation. The identified categories in literature are considered to be a baseline, to identify factors as elements of a generic holistic implementation model or protocol for new technological equipment in ORs.

## 1. Introduction

Operating rooms (ORs) are complex technological environments and high-reliability organisations (HROs), in which technological equipment and information technology are used to perform (surgical) procedures [1–4]. Advancements and innovations in medical technology continue, which result in frequent implementations of new technological equipment in ORs. According to Edmondson [5], the implementation of new technological equipment entails the integration of technology in day-to-day activities in an organization [5]. In order to ensure the safe use of medical technology, the Dutch Hospital Association (DHA) agreed upon a set of policies published in the Covenant Medical Technology (CMT). The CMT states that hospitals should have defined and implemented safety policies regarding medical technological equipment. Compliance to these policies is audited by the Dutch Health and Youth Care Inspectorate (HYI) [4, 6]. These policies involve acquiring, implementing, using, and disposing medical equipment. To comply with the CMT, hospitals have defined hospital specific local policies to implement new medical technological equipment. These local policies result in local procedures to implement new technological equipment, resulting in varying implementation activities, lead times, and success of implementations. We postulate that these variations result in inefficiencies and cause lower adaptation rates due to difficulties with the integration of new technological equipment in day-to-day activities and thus in clinical practice. Moreover, in contrast to the strictly regulated introduction of new drugs provided by the pharmaceutical industry, generic detailed guidelines for the implementation of medical technology do not exist. Within the field of information sciences, the success of implementations of information technology (IT) has increased and some scholars identify factors for successful implementations of IT for instance technological factors, organisational factors, and job factors [7, 8]. However, much remains unexplored, especially when considering all these perspectives holistically. The overall aim of our research is to develop a holistic model for implementation of new equipment in ORs, which helps hospitals and medical equipment companies to implement medical technology in a safe, efficient, and cost-effective way. For reasons of demarcating and focus, we concentrate on the implementation of new medical technological equipment, which includes medical equipment and medical information technology (i.e., hardware and/or embedded software). This study is the first step towards our overall aim, and we analyze existing recent literature available on implementations of technology in the OR, in order to identify success factors for efficient implementations. Results from this study will be included in the development of a holistic implementation model for new technological equipment in ORs. In the following section, we explain the literature search and analysis procedure, followed by a section that describes the literature review results. In the discussion, we reflect on the results. In the last section conclusions, limitations and plans for further research are provided.

## 2. Method

**Figure 1:**
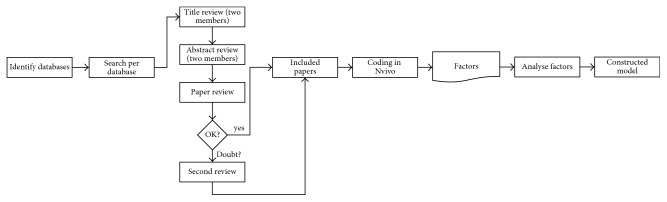
Overview of search activities and coding.

The aim of our systematic literature review is to identify all types of relevant factors on the implementation of medical technology in ORs and to categorize these factors. To ensure quality and rigor, this systematic literature review commenced by setting up a literature search protocol following the guidelines of Kitchenham and Charters (Figure 1) [9]. The following databases were accessed in the search process: Academic Search, ACM, DOJ, Embase, NARCIS, Pubmed, Science Direct, Springerlink, Web of Science, and Wiley. We entered the following terms and operators: “Implement” OR “Implementation” AND “Technology” AND “Operating Room.” These terms were searched for in “all fields” of selected databases.

### 2.1. Inclusion and Exclusion Criteria

We used no date restrictions during the database search. Articles regarding the implementation of medical equipment as well as information technology were included in the reviewing process. Titles of articles included in reference lists related to the search criteria were considered. Articles published in other than the English language were excluded. We excluded secondary literature, for example, books. Conference abstracts, poster presentations, and letters were excluded as well, due to limited availability of detailed information in proceedings and other sources.

We reviewed the results in three steps. Firstly, two members of the research team (NSM and BVZ) reviewed titles independently according to predefined inclusion and exclusion criteria. Titles with positive reviews by the two researchers were included for the abstract review; titles with a negative and positive review by the researchers were included in the abstract review; and titles with double-negative reviews were excluded for the abstract reviews (NSM and BVZ). Secondly, we reviewed abstracts independently and similar to the title review process (NSM and BVZ). Abstract review results were discussed, resulting in a selection of abstracts for full-article review. Duplicate abstracts were removed. In the third phase, the selection of full articles was reviewed for inclusion or exclusion, according to the purpose of the research (NSM). In case of doubt, the second reviewer (BVZ) was asked to assess the article. Results of the full-article review were discussed, and articles were in- or excluded by consensus.

### 2.2. Coding

Coding of included articles should be resulting in all types of influencing factors for the implementation of medical equipment in ORs as well as resulting factors of an implementation of medical equipment for instance performance. During a coding process, relevant sections in articles are marked and a descriptive name or code is added to the section. During coding of included articles, all relevant sections were coded inductively using NVivo (version 11 for Windows) [10]. Through “open coding,” we identified factors or categories of importance in our literature sources, following principles as presented by Strauss and Corbin and leveraging Nvivo tooling [11].

## 3. Results

### 3.1. Search Results

**Figure 2:**
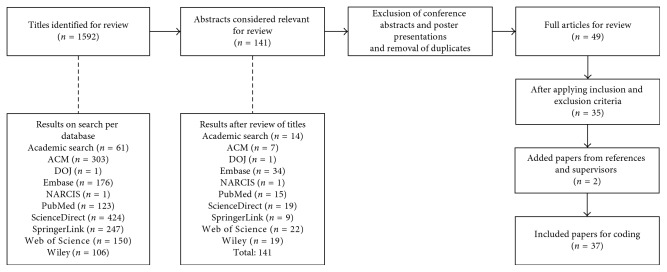
Search results.

Our searches resulted in 1592 potentially eligible articles (Figure 2). After screening titles, 1451 articles were excluded. After reviewing the abstracts of 141 studies, 49 articles remained. Reviewing these articles and applying the inclusion and exclusion criteria resulted in 35 remaining articles for detailed coding and analysis. Two articles were added to this selection based on references and feedback from coresearchers, that is, Raman et al. and Stefanidis et al. [12, 13]. During the search and coding process, the article of Raman et al. was an accepted, not yet published, manuscript. This article provided insights into the implementation of checklists in OR and was therefore included in this research. The second article from Stefanidis et al. was published as a set of guidelines for the introduction of new technology and techniques from a surgeons' perspective. This article did not include an abstract nor keywords that were related to this research. The research team advised to include these articles due to their relevance and the scope of this research.

Following the review process and criteria for inclusion and exclusion, 37 articles were included in this study.

Table 1 provides an overview of included articles related to the year of publication, with intervals of 5 years. Three included articles were published in interval I, period 1997–2002. Six articles were published in the periods referring to intervals II and IV. Most included articles (*n* = 22) were published in interval III, corresponding to the period 2009–2014.

### 3.2. Coding Results

The coding process resulted in a long list of descriptive names or items. Related items were grouped in categories or factors. This process is traceably and transparently performed in NVivo.

Table 2 shows seven categories that are derived from the coded items: communication, performance, process and activities, project management, staff, technology, and training. Each category consists of one or more underlying items, resulting from coding articles in NVivo. Furthermore, Table 1 shows the number of coded articles per category (“number of articles”). The categories process and activities, staff, and technology are referenced in the majority of the coded articles, respectively, 29, 30, and 27 articles. Table 2 also shows the aggregated frequency of coded items per category (“aggregated frequency of coding”). Based on the aggregated frequency of items, the categories project management, technology, and process and activities, are coded most often, respectively, 510, 355, and 240 times. These results imply that underlying items of these categories are coded more than once in corresponding articles.

The identified categories are explained in the following sections:

### 3.3. Communication

Communication is a category that was coded in 24 articles. When new technology (i.e., medical equipment) is introduced, disruptions in activities and workflow occur, which require communication and teamwork. Communication with relevant stakeholders is one of the factors to prevent errors when introducing new technological equipment. The use of updated checklists is described as one of the communication tools, which regulate activities and the workflow for stakeholders such as surgeons, anaesthesiologists, and surgical supporting staff. The use of these updated checklists contributes to improved safety in the OR [12–34].

### 3.4. Performance

In 22 articles, various indicators regarding performance are identified such as OR efficiency and performance, patient care, patient outcomes, finance, safety, ergonomics, and user-friendliness of technological equipment [5, 12, 16, 19, 21–24, 26–32, 35–41].

### 3.5. Processes and Activities

The majority of the articles included in this study showed that the introduction of new technological equipment affects processes and activities of employees in the OR. Tasks and activities of OR employees are recorded in protocols and checklists to ensure safety and quality in pre-, per- and postoperative activities of surgeries. Task deconstructions of involved employees are used to analyze the impact of a new device on performed activities, processes, and workflows. Alterations in processes and workflows result in updated protocols and checklists, affecting tasks and activities for involved employees [5, 12–18, 21–32, 34, 36–40, 42–46].

### 3.6. Project Management

In the OR, many stakeholders are involved, executing various protocolled tasks and activities. Implementation of new technological equipment as a project requires management to achieve predetermined goals. Identified elements for project management regard the identification of stakeholders, defining the purpose of the project, as well as benefits and gains. A project plan and planning are considered to be part of this category. During the process of implementation, team members are identified to execute a project plan. Multiple articles mention the allocation of a multidisciplinary team as one of the necessary factors for the implementation of new technology, as different perspectives to the implementation are addressed. Examples of these perspectives are change management, simulations, and stakeholder management [12, 14–18, 20–23, 25–27, 29–31, 33–35, 37–43, 47].

### 3.7. Staff

When referred to as staff in the OR, we refer to employees or surgical supportive staff who are involved in setting up, preparing, using, and disassembling medical equipment. The ease of use of new medical equipment contributes to the adoption of this equipment by staff. During the project, staff need to be involved in activities regarding the new equipment, such as training, setting up, using, and disassembling medical equipment and updating corresponding protocols and checklists [5, 12, 13, 15, 16, 18–27, 29, 30, 32–37, 39–41, 48].

### 3.8. Technology

In this review, technology is used as category for coding referring to medical equipment and (embedded) Information Technology (IT). Studies show that the implementation of new medical equipment involves integrating new technology in the daily processes and activities. Relevant training for staff is required which includes setup, use, disassembly of equipment, the interpretation of data and screens (if applicable), and troubleshooting in case problems occur [5, 12, 13, 15–20, 22, 24–26, 28, 29, 31, 32, 34, 36–38, 40, 42–45, 47].

### 3.9. Training

Studies showed that staff needs training to setup, configure, use, and disassembly new medical equipment. Training starts during the project with involved project members and based on the project plan and product requirements. Training elements are described in training programs, which entail technical and nontechnical skills. Nontechnical skills are described as skills regarding communication, teamwork, and leadership. Depending on the contents of training, staff gain experience and skills to use medical equipment and to interpret data (on screens if applicable). Skills to troubleshoot when problems occur are needed as well. Based on the type of equipment and corresponding risks, manufacturers and educators should define ways of (ongoing) training assessment [5, 12, 15, 17–19, 21, 22, 25–30, 32–34, 37–42, 44, 46].

## 4. Discussion

There is overwhelming evidence that the use of medical technology and information technology in ORs will increase. This will affect costs, quality of care, complexity of surgical procedures and, as a consequence, also patient safety. Current guidelines, available for implementation of new devices in the OR, in essence include safety based on the local policies according to the covenant medical technology in the Netherlands (CMT) [4]. The OR is a dynamic, multidisciplinary, multistakeholder, and innovative environment, and the development and implementation of medical equipment should not only consider safety but also cost and effects. Although the CMT policy represents a guideline for local hospitals and audits are performed by the Dutch Health and Youth Care Inspectorate (HYI), we learned from literature and experience that implementation of new medical equipment runs along all different sorts of pathways before being accepted in clinical surgical practice. We also learned that implementations vary in duration and success. In this review, coded seven main categories are indeed relevant, and all have their impact in the process of implementation: “processes and activities,” “staff,” “communication,” “project management,” “technology,” “training,” and “performance.” Table 1 shows that the number of referenced articles varies between 22 articles and 30 articles out of a total of 37 articles. The aggregated frequency of coding shows that the categories project management, technology, and processes and activities are referenced 510, 255, and 240 times. Prior to the coding process, we expected that implementations of new technologies effected processes and activities, technology, and staff; this is indeed confirmed by literature. Results show that the category project management scores high, due to the accumulation of frequencies of underlying coded items. These items are expected to be part of an integral implementation project of new medical equipment, consisting of various project activities.

We postulate that aforementioned categories provide a baseline for a holistic perspective on implementations of new medical equipment in ORs. The category performance can be identified as a resulting category related to the outcome of an implementation, while the other categories can be identified as influencing categories. We further postulate that tailoring or aligning these influencing categories and underlying items to the context such as organisation, type of medical equipment, or involved stakeholders affect the outcome of an implementation.

Based on this literature review and our logistical and clinical experience, we will focus on the alignment of the factors “technology,” “processes and activities,” and “staff” to improve the success of implementations of medical equipment.

## 5. Conclusions and Further Research

Development and implementation of innovative medical equipment to improve safety, quality, or efficiency are common practices in hospitals all around the world. Integral guidelines for implementation of new medical equipment are not yet available. This literature review shows that six main influencing categories can be identified based on the selected studies: “processes and activities,” “staff,” “communication,” “project management,” “technology,” and “training;” the anticipated outcome of implementations is identified as the resulting category “performance.” As the integration of new technology in daily activities remains a challenge, we will develop a generic holistic model for implementations of medical equipment in ORs guided by the results of this literature review. The identified categories are considered to be a baseline, which identifies influencing factors as elements of a generic holistic implementation model for new technological equipment in the OR. We suggest that this model is based on the alignment of the identified categories and the medical equipment to be implemented. Principles from strategic alignment in Information Systems research are considered to be a promising approach for developing a model: aligning technology introduction with organizational processes and organization strategy [49].

This study focused only on written scientific sources in hospitals or ORs and therefore probably omits certain aspects that may become visible through performed case studies. We are conducting explorative case studies and anticipate that these studies will contribute in developing specific and reproducible routes for implementation of medical equipment and thus add other relevant categories to those we identified in literature. We expect that a model for implementation of medical equipment in ORs provides insights into various stakeholders and companies and that this model will enable various stakeholders in hospitals to implement new technological equipment in a generic way in ORs, contributing to further enhanced safety as well as efficiency and to shorten the duration of the implementation process.

## Figures and Tables

**Table 1:** Results: distribution of articles according to the year of publication.

Interval	Period (year)	Number of articles (*n* = 37)
I	1997–2002	3
II	2003–2008	6
III	2009–2014	22
IV	2015–2016	6

**Table 2:** Results: frequencies of coded categories.

Legend	Categories/factors	Number of articles	Aggregated frequency of coding
1	Communication	24	86
2	Performance	22	86
3	Process and activities	29	240
4	Project management	24	510
5	Staff	30	190
6	Technology	27	355
7	Training	25	176
